# Advancements in Information-Theoretic Methods for Data Analytics

**DOI:** 10.3390/e27070708

**Published:** 2025-06-30

**Authors:** Kichun Lee

**Affiliations:** Department of Industrial Engineering, College of Engineering, Hanyang University, Seoul 133-791, Republic of Korea; skylee@hanyang.ac.kr

## 1. Introduction

A defining feature of the current epoch is a historically unparalleled increase in information, data that originates from a wide spectrum of scientific disciplines, industrial processes, and societal interactions [1,2]. Such rapid growth in data volume gives rise to substantial prospects alongside considerable hurdles, chiefly revolving around the ability to extract valuable understanding and practical intelligence from extensive and frequently intricate data collections [3,4]. Successfully maneuvering through this data-abundant landscape requires the design and deployment of advanced analytical structures able to reveal latent regularities, assess levels of ambiguity, and support well-grounded choices [2,5].

At the core of these analytical methods are information-theoretic methods. Based on the fundamental principles established by Shannon and subsequent researchers, information theory provides a mathematical framework for quantifying information, characterizing uncertainty through measures such as entropy, and understanding the fundamental limitations of data compression, communication, and inference [6,7]. These methods have proven useful as the basis for many modern techniques in data mining, machine learning, statistical inference, and pattern recognition [7,8]. As an essential tool for understanding raw data, information-theoretic approaches to modeling complex dependencies, selecting features, and guiding the development of robust algorithms are used in numerous areas of information theory [8,9].

The field of information-theoretic data analysis is undergoing continuous research and development, with the goal of improving the efficiency and accuracy of existing methods and developing approaches to solve new problems. Key areas of research include improving information measures [10], developing robust estimation techniques for high-dimensional and noisy data, and developing new algorithms for tasks such as feature selection [9], clustering [11], and detecting anomalies. The integration and development of information theoretic concepts and computational tools, including deep learning and artificial intelligence, that can capture more sophisticated and nuanced features and interpretations of complex phenomena is advancing research while further enhancing the capabilities of data analytics [8,12,13].

This Special Issue, entitled “Information-Theoretic Methods in Data Analysis,” presents a collection of 12 peer-reviewed papers that reflect the diversity of methods and applications in this area of research. These papers present new information-based methodologies and their various applications, with particular emphasis on the development of robust statistical frameworks and the study of information flow within complex systems. In addition, they cover a wide range of topics ranging from fundamental theoretical developments to new estimation procedures and innovative and appropriate applications of information theory principles in fields such as medicine, finance, engineering, and natural sciences.

This Editorial aims to provide an overview of the papers in this Special Issue, summarize the main contributions, and highlight the importance of information-theoretic data analysis to various fields.

## 2. Contributions to ‘Information-Theoretic Methods in Data Analytics’

The 12 articles compiled in this Special Issue offer a compelling panorama of the current research landscape at the intersection of information theory and data analytics. Collectively, these papers demonstrate the remarkable adaptability and profound utility of information-theoretic concepts in tackling a wide array of contemporary challenges, ranging from fundamental methodological advancements to specific, impactful applications. For a structured overview, Table 1 summarizes the key aspects of each contribution. The ensuing discussion will further elaborate on the insights and advancements presented by these works, thereby underscoring the importance of this collection.

Broadly, the contributions in this Special Issue revolve around three interconnected themes: firstly, (1) advancing foundational information-theoretic methodologies and their estimation, particularly for complex data scenarios; secondly, (2) information theory in machine learning, enhancing models, and its applicability and understanding; and thirdly, (3) applications of information-theoretic methods in diverse data domains.

### 2.1. Advancing Foundational Methodologies and Estimation

Recent research has developed methods to advance information theoretical measurement methods. There are problems such as high-dimensional data, errors in measurement, and sequential data dependence, and research has focused on the development of robust estimation techniques that address such issues.

Yu (Contribution 8) deals with the pervasive measurement error problem in high-dimensional regression analysis. The proposed Adaptive CoCoLasso method combines a recent positive quasi-verbal projection with an adaptively weighted $l_{1}$ penalty. This methodology achieves better balance between performance and efficiency, which is crucial, especially in situations where noise makes actual key information unclear. This strategy, which follows an information-based regularization framework, seeks to achieve efficient and reliable information extraction, the goal of information theory, by prioritizing signal-to-noise features.

Addressing the challenge of understanding complex dependencies in sequential data, Tsur and Permuter (Contribution 11) introduce their work in “InfoMat: Leveraging Information Theory to Visualize and Understand Sequential Data”. As a significant advancement in the understanding and utilization of information flow within dynamic systems, a new visualization tool based on conditional mutual information called ’InfoMat’ is introduced. By representing how information is delivered in a sequential system in a structured visual form, InfoMat clearly shows key measurements such as directional information or transfer entropy. To ensure applicability to real-world data, the authors developed an efficient Gaussian mutual information estimator, as well as a neural InfoMat estimator based on masked auto-regression. This study is significant in that it connects quantitative figures with human-interpretable patterns, transforming abstract and sometimes elusive information theoretical measurements into intuitive visual representations. This visualization will contribute to the popularization and in-depth understanding of information flow analysis techniques.

In a related exploration of system dynamics, in “Detecting Signatures of Criticality Using Divergence Rate,” Chan, Soh, and Hillar (Contribution 12) investigate “signatures of criticality”—points at which complex systems undergo dramatic changes, often associated with optimal information processing. Their proposed “divergence rate” measure, grounded in Kullback–Leibler (KL) divergence and guided by rate-distortion (RD) theory, quantifies the rate of change in a system’s behavior as a control parameter is varied. Peaks in this divergence rate are identified as critical control settings. The application of RD theory and KL divergence to detect these critical points suggests a profound connection: optimal system states, in terms of information processing efficiency or adaptation, might inherently reside at these transitional “edges of chaos,” where the trade-off between information compression (rate) and fidelity (distortion) is critically balanced.

### 2.2. Information Theory in Machine Learning: Enhancing Models and Understanding

To improve the performance of machine learning models, refine them, and better understand how they work, researchers in recent years have been leveraging information theoretic concepts. In this Special Issue, many of the papers showcase research that utilizes and applies the principles of information theory in a variety of ways.

Zhang et al. (Contribution 1) use information gain as a key way to identify topics in the literature related to text sensitivity analysis in “Topical Discovery and Hotspot Analysis of Sentimental Information-Theoretical Method.” Their model calculates the information gain of keywords associated with various topics, identifying the characteristic words that most clearly distinguish one topic from another. This process effectively quantifies how much uncertainty there is in classifying a topic for a specific keyword. This direct quantification of feature discrimination allows for the better organization and improvement of existing heuristic approaches used in text analysis and, consequently, a deeper understanding of research trends and hotspots. In addition, by integrating FastText and hierarchical clustering for word embeddings, we contribute to building a robust pipeline for literature analysis.

The paper “Anomaly Detection Using an Ensemble of Multi-Point LSTMs” by Lee, Yoon, and Lee (Contribution 3), while primarily focusing on LSTM-based ensemble learning, operates within a problem space deeply connected to information theory. Anomaly detection can be framed as identifying data points that are “surprising,” possessing high “information content” or low probability under an established model of normal behavior, thereby exhibiting high self-information. The proposed LSTM ensemble, which combines various predictive models, seeks to build a stronger definition of ’normality’. As a result, real anomalies that deviate from the learned normal category become statistically clearer, consistent with the principle of identifying phenomena with high Shannon information.

Li, Dong, and Li (Contribution 6), in “A Study of Adjacent Intersection Correlation Based on Temporal Graph Attention Network,” utilize information gain to identify the most influential traffic features (such as average delay) for classifying intersection states. Their Temporal Graph Attention Network (TGAT) model simultaneously classifies states and calculates inter-intersection correlations. Applying information gain as a principled methodology for feature engineering in this complex space–time domain allows us to quantify which parameters carry the most important information about the intersection state or reduce the uncertainty the most. In their paper, titled “Motor Fault Diagnostics Based on Convolutional Block Attraction Module-Xception Lightweight Neural Network,” Xie et al. (Contribution 7) use the Gram Angle Field (GAF) image coding scheme to convert one-dimensional oscillation signals into two-dimensional images for defect diagnosis. The convolutional block attention module (CBAM) selectively amplifies the most prominent or informative features within the two-dimensional representation, further refining the entire transformation process. The two-dimensional representations targeted for such elaboration are obtained through transformations encoding temporal region information, which can be understood as clarifying correlations with temporal patterns that may have been difficult to grasp in the original one-dimensional data, potentially further enhancing mutual information between input signal representation and defect states in subsequent neural network classifiers.

Wang et al. (Contribution 9) deal with the feature selection problem in their paper, titled “Dual-Regularized Feature Selection for Class-Specific and Global Feature Associations.” Their proposed Dual-Regularized Feature Selection (DRFS) method incorporates both class-specific feature manifold conservation and a regularizer for global feature deduplication. The methodology itself uses RBF kernels and Laplacian matrices, but it essentially works on the principle of selecting the “most informative features.” This approach implicitly aims at maximizing the relevance of features to class labels (similar to mutual information maximization), considering conditional relevance within specific classes, and suppressing redundant information between selected features (similar to combined entropy minimization or conditional entropy maximization).

### 2.3. Applications of Information-Theoretic Methods in Diverse Data Domains

The versatility of information-theoretic tools is further underscored by their application across a wide range of data types and problem domains, as demonstrated by several articles in this Special Issue. These applications often leverage information-theoretic concepts either directly or as an underlying principle for data interpretation and model design.

Li et al. (Contribution 2), in “Sentiment Analysis on Online Videos by Time-Sync Comments,” develop a model to analyze sentiment in video highlights using time-sync comments (TSCs). A key component of their highlight score is TSC density ($D_{fv,n}$), which represents the number of comments at a specific point in a video. This density can be interpreted as an implicit measure of “surprise” or high “information content” from the audience’s perspective, where segments eliciting a higher volume of comments are likely those that deviate from passive viewing, indicating heightened engagement or emotional response that signifies informative moments. This approach cleverly uses user-generated data as a proxy for identifying segments with high informational salience.

The work by Zhang et al. (Contribution 4) in “Information Difference of Transfer Entropies between Head Motion and Eye Movement Indicates a Proxy of Driving” provides a direct and compelling application of transfer entropy (TE) to understand human behavior. By quantifying directed information flow—specifically, the reduction in uncertainty for one time series given the past of another—between head motion and eye movements, the authors establish that in goal-directed driving, head motion tends to lead eye movement predictively. More importantly, the normalized one-way information difference (NUID) derived from this TE shows a correlation with driving performance. This work demonstrates the power of TE in identifying causal effects within complex systems by linking abstract cognitive processes with quantifiable information-theoretic measures.

In the domain of advertising analytics, Arslan et al.’s (Contribution 5), titled “A Comprehensive Framework for Measuring the Immediate Impact of TV Advertisements: TV-Impact,” presents a framework that leverages CausalImpact, a Bayes structural time series model, to decouple the effects of TV advertising on online traffic. A fundamental challenge is the problem of information disentanglement: determining how much of the observed traffic growth (information) is due to TV advertising and how much of it is due to other simultaneous factors. The new dynamic control variable selection method and “Group Ad” influencing the decomposition method presented by this framework contribute to the finer attribution of this information impact.

Finally, in “AlphaRouter: Bridging the Gap Between Reinforcement Learning and Optimization for Vehicle Routing with Monte Carlo Tree Searches,” Kim et al. (Contribution 10) introduce an innovative application of an entropy-based strategy within a reinforcement learning context. AlphaRouter selectively applies Monte Carlo Tree Search (MCTS) based on the uncertainty (Shannon entropy) of the policy network’s output distribution. When the policy network is highly uncertain about the next best action (i.e., the entropy of its output probability distribution is high), MCTS is invoked for a more thorough search. This can be viewed as an information-theoretical control mechanism for computation, similar to metacognitive strategies in which agents optimize the use of information resources by deciding whether to rely on faster “intuitive” (direct policy output) searches or performing more effort-intensive “considered” MCTSs.

Collectively, these 12 papers clearly and convincingly demonstrate that information theory is not merely a theoretical discipline but an active and evolving source of practical tools and conceptual frameworks for data analytics. The recurring themes of quantifying uncertainty, measuring information flow, identifying relevant features, and understanding complex dependencies highlight the fundamental contributions of information-theoretic principles. As datasets continue to grow in size and complexity, and as the demand for interpretable and robust analytical solutions intensifies, information theory is poised to become even more central to the advancement of data science.

## Figures and Tables

**Table 1:** An overview of the papers in the Special Issue “Information-Theoretic Methods in Data Analytics”.

List of Contrihbutions	Title	Authors	Keywords	Core Contribution
1	Topic Discovery and Hotspot Analysis of Sentiment Analysis of Chinese Text Using Information-Theoretic Method	Zhang et al.	Text sentiment analysis, NLP	Proposes an Information Gain-based model for topic discovery in sentiment analysis literature
2	Sentiment Analysis on Online Videos by Time-Sync Comments	Li et al.	Video sentiment analysis	Proposes a DTSC-based model for video sentiment recognition using TSC density for engagement
3	Anomaly Detection Using an Ensemble of Multi-Point LSTMs	Lee et al.	Time-series anomaly detection	Proposes an ensemble of multi-point LSTMs for robust time-series anomaly detection
4	Information Difference of Transfer Entropies between Head Motion and Eye Movement Indicates a Proxy of Driving	Zhang et al.	Human behavior analysis, driving	Quantifies eye–head coordination using transfer entropy (TE); NUID derived from TE correlates with driving performance
5	A Comprehensive Framework for Measuring the Immediate Impact of TV Advertisements: TV-Impact	Arslan et al.	Advertising analytics, causal inference	Introduces TV-Impact framework with CausalImpact for measuring TV adverts’ effects on online traffic
6	A Study of Adjacent Intersection Correlation Based on Temporal Graph Attention Network	Li et al.	Traffic management, urban computing	Proposes a TGAT-based model for traffic state classification and intersection correlation using Information Gain
7	Motor Fault Diagnosis Based on Convolutional Block Attention Module-Xception Lightweight Neural Network	Xie et al.	Industrial fault diagnosis	Develops a CBAM-Xception network with Gram-coded signals for motor fault diagnosis
8	Adaptive CoCoLasso for High-Dimensional Measurement Error Models	Yu, Q	High-dimensional regression	Introduces Adaptive CoCoLasso for robust estimation in high-dimensional linear models with measurement errors
9	Dual-Regularized Feature Selection for Class-Specific and Global Feature Associations	Whang et al.	Feature selection, ML	Proposes DRFS with dual regularizers for class-specific manifold preservation and global redundancy elimination
10	AlphaRouter: Bridging the Gap Between Reinforcement Learning and Optimization for Vehicle Routing with Monte Carlo Tree Searches	Kim et al.	Reinforcement learning, optimization	Presents AlphaRouter combining DRL with MCTS, which is selectively applied based on policy network entropy
11	InfoMat: Leveraging Information Theory to Visualize and Understand Sequential Data	Tsur et al.	Sequential data analysis, visualization	Introduces InfoMat, a matrix for visualizing information transfer in sequential systems using Conditional Mutual Information
12	Detecting Signatures of Criticality Using Divergence Rate	Chan et al.	Complex sSystems, ML, NLP	Proposes a Divergence Rate measure (KL Divergence and RD Theory-based) for detecting critical phase transitions
